# 5G-AKA-FS: A 5G Authentication and Key Agreement Protocol for Forward Secrecy

**DOI:** 10.3390/s24010159

**Published:** 2023-12-27

**Authors:** Ilsun You, Gunwoo Kim, Seonghan Shin, Hoseok Kwon, Jongkil Kim, Joonsang Baek

**Affiliations:** 1 Department of Financial Information Security, Kookmin University, Seoul-si 02707, Republic of Korea; isyou@kookmin.ac.kr (I.Y.); gguakim22@kookmin.ac.kr (G.K.); hoseok1997@kookmin.ac.kr (H.K.); 2 Cyber Physical Security Research Center, National Institute of Advanced Industrial Science and Technology (AIST), Tokyo 135-0064, Japan; 3 Department of Cyber Security, Ewha Womans University, Seoul-si 03760, Republic of Korea; jongkil@ewha.ac.kr; 4 School of Computing and Information Technology, University of Wollongong, Northfields Avenue, Wollongong, NSW 2522, Australia; baek@uow.edu.au

**Keywords:** 5G security, 5G-AKA, forward secrecy (FS), standard compatibility

## Abstract

5G acts as a highway enabling innovative digital transformation and the Fourth Industrial Revolution in our lives. It is undeniable that the success of such a paradigm shift hinges on robust security measures. Foremost among these is primary authentication, the initial step in securing access to 5G network environments. For the 5G primary authentication, two protocols, namely 5G Authentication and Key Agreement (5G-AKA) and Improved Extensible Authentication Protocol Method for 3rd Generation Authentication and Key Agreement (EAP-AKA′), were proposed and standardized, where the former is for 3GPP devices, and the latter is for non-3GPP devices. Recent scrutiny has unveiled vulnerabilities in the 5G-AKA protocol, exposing it to security breaches, including linkability attacks. Moreover, mobile communication technologies are dramatically evolving while 3GPP has standardized Authentication and Key Management for Applications (AKMA) to reuse the credentials, generated during primary authentication, for 5G network applications. That makes it so significant for 5G-AKA to be improved to support forward secrecy as well as address security attacks. In response, several protocols have been proposed to mitigate these security challenges. In particular, they tried to strengthen security by reusing secret keys negotiated through the Elliptic Curve Integrated Encryption Scheme (ECIES) and countering linkability attacks. However, they still have encountered limitations in completing forward secrecy. Motivated by this, we propose an augmentation to 5G-AKA to achieve forward security and thwart linkability attacks (called 5G-AKA-FS). In 5G-AKA-FS, the home network (HN), instead of using its static ECIES key pair, generates a new ephemeral key pair to facilitate robust session key negotiation, truly realizing forward security. In order to thoroughly and precisely prove that 5G-AKA-FS is secure, formal security verification is performed by applying both BAN Logic and ProVerif. As a result, it is demonstrated that 5G-AKA-FS is valid. Besides, our performance comparison highlights that the communication and computation overheads are intrinsic to 5G-AKA-FS. This comprehensive analysis showcases how the protocol effectively balances between security and efficiency.

## 1. Introduction

Since the first deployment of the fifth generation (5G) mobile networks in 2019, 5G has rapidly become a mainstream mobile network worldwide. According to the Global System for Mobile Communications Association (GSMA), the 5G adoption rate reached 43.1% in the United States in Q4 2022 [1]. 5G mobile networks and telecommunication standards have been developed to meet the various demands of modern telecommunication. In particular, it is designed to support enhanced features such as Enhanced Mobile BroadBand (EMBB), Massive Machine-Type Communications (MMTC), and Ultra-Reliable and Low-Latency Communications (URLLC) [2].

**Figure 1:**
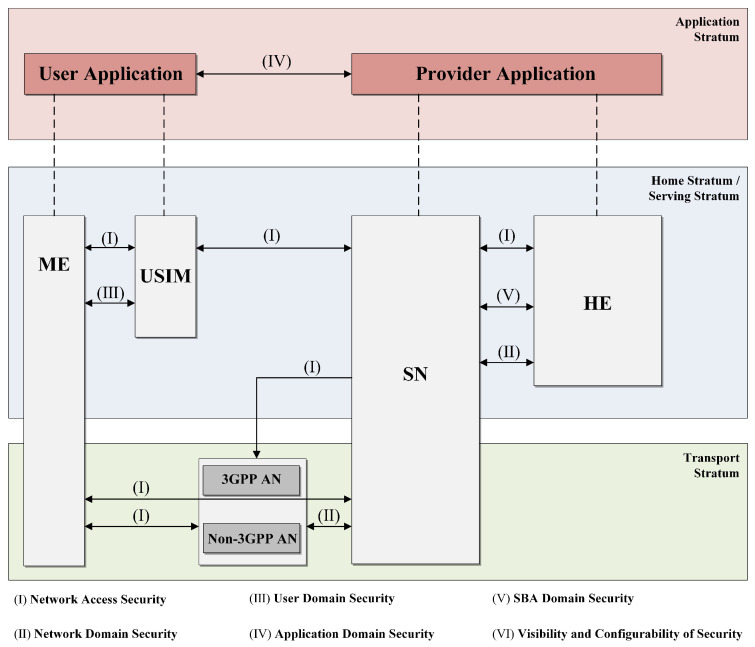
3GPP 5G security architecture.

In order to ensure the seamless delivery of high-quality services to users through these three features, 5G demands heightened security compared to its predecessors in mobile networks. Therefore, the 3rd Generation Partnership Project (3GPP) consortium, which is in charge of the standardization of 5G mobile networks, has standardized an essential 5G security architecture and procedures as well as several security features [3,4]. Figure 1 depicts the 3GPP 5G security architecture.

Notably, in 5G, primary authentication emerges as a cornerstone, representing the initial security checkpoint for accessing 5G network environments. For the 5G primary authentication, two protocols have been adopted as standards: 5G Authentication and Key Agreement (5G-AKA) and Improved Extensible Authentication Protocol Method for 3rd Generation Authentication and Key Agreement (EAP-AKA${}^{\prime }$) [3,5]. The former caters to 3GPP devices, while the latter is tailored for non-3GPP devices.

The 5G primary authentication protocols, i.e., 5G-AKA and EAP-AKA${}^{\prime }$, allow a user equipment (UE) (e.g., a mobile phone) and a home network (HN) (e.g., the network of a service provider) to authenticate each other and exchange key materials (e.g., anchor keys) to protect entire subsequent 5G communications. Note that they have been enhanced and markedly differentiated from their previous version (i.e., Evolved Packet System Authentication and Key Agreement (EPS-AKA) [4] and Extensible Authentication Protocol Method for 3rd Generation Authentication and Key Agreement (EAP-AKA) [6]). The most distinctive feature of those security protocols in the 5G network is using a Subscriber Concealed Identifier (SUCI) that can be explained as a Subscriber Permanent Identifier (SUPI) in the encrypted format. In the previous generation, authentication was performed by transmitting the user identification information, International Mobile Subscriber Identity (IMSI), without encryption. On the other hand, in 5G, SUPI, which is a UE’s identifier, is encrypted into SUCI using a key derived through the Elliptic Curve Integrated Encryption Scheme (ECIES) before transmission to address identifier exposure [7,8,9,10]. Despite these efforts, 5G-AKA still remains vulnerable to various types of attacks [7,9,10,11,12,13,14]. Ref. [15] described vulnerabilities for 5G-AKA through formal verification and analysis. The authors showed that there still exist attack scenarios against 5G-AKA. In addition, refs. [10,16] pointed out that privacy problems of users may occur since 5G-AKA is susceptible to linkability attacks. Furthermore, ref. [17] presented shortcomings of 5G-AKA, including the lack of support for forward secrecy, also known as perfect forward secrecy.

Meanwhile, the 5G network environments face the following new challenges:

- As the advancement of 5G technology and the proliferation of 5G network applications continue at a rapid pace, the emergence of new security threats and attacks becomes more pronounced, thereby necessitating the establishment of elevated security prerequisites.
- Thanks to the Authentication and Key Management for Applications (AKMA) [18], the credentials generated through 5G primary authentication can be reused for application authentication in the 5G network environments; that is, the master session key negotiated during the 5G primary authentication is applied to derive an application key that allows a UE to authenticate itself to AKMA-based applications smoothly and efficiently.

Evidently, the security robustness of the existing 5G primary authentications falls short of addressing the aforementioned concerns. As a result, it becomes imperative to bolster the security framework with robust public key-based measures and ensure the implementation of forward secrecy.

Regarding EAP-AKA${}^{\prime }$, there has been a standardization effort aimed at enhancing the protocol to incorporate support for forward secrecy (known as EAP-AKA${}^{\prime }$-FS) [19]. On the 5G-AKA side, there are existing works proposed to support unlinkability and forward secrecy, such as [3,16,17,20,21]. In particular, those 5G-AKA enhancements attempted to reuse the ECIES shared key, which is used to protect the UE’s identifier in the initiation phase. However, in such an approach, if an adversarial security event happens in HN, forward secrecy is not guaranteed for the session key.

In this paper, we propose a 5G-AKA-Forward Secrecy (5G-AKA-FS) protocol that supports forward secrecy and unlinkability together to solve the limitations of the existing studies and maximize the efficiency of authentication in 5G networks. The proposed protocol accomplishes forward secrecy and unlinkability by introducing an additional ECIES-based ephemeral key pair generation within HN. The main contributions of this paper are summarized as follows:

- The 5G-AKA-FS protocol is designed to concurrently support forward secrecy and unlinkability.
- We analyze the latest studies proposed to improve the vulnerabilities of 5G-AKA and provide a solid comparison of security attributes between our 5G-AKA-FS and the latest studies.
- We conduct a rigorous security verification of the proposed protocol using formal verification (BAN Logic [22] and ProVerif [23]).
- Performance evaluation is thoroughly carried out by measuring overhead in terms of computation and communication.

The rest of the paper is organized as follows. Section 2 explores the existing enhancements of 5G-AKA, while Section 3 describes the preliminaries used in this paper. Moving on to Section 4, we present a detailed description of the proposed protocol, followed by the formal security analysis using BAN Logic and ProVerif in Section 5. Assessing performance, Section 6 carries out a comparative evaluation of security properties between the proposed and existing protocols, along with measured overhead. Finally, Section 7 provides the conclusion.

## 2. Related Works

3GPP has defined two AKA protocols, namely 5G-AKA and Extensible Authentication Protocol (EAP)-AKA${}^{\prime }$, for primary authentication in 5G networks. The purpose of the AKA protocols is to establish mutual authentication between the UE and the HN. In 2G to 4G networks, UE’s identifier IMSI is publicly exposed, thus resulting in privacy issues. However, in 5G-AKA, the subscriber identity information, known as SUPI, is encrypted into SUCI, which is then used for authentication while solving the privacy problem.

**5G-AKA** enhances authentication and key exchange in addition to introducing subscriber identity protection (e.g., SUCI) for privacy. However, as it is developed based on EPS-AKA, it inherits existing security vulnerabilities. Ref. [15] conducted a formal verification and analysis of the 5G-AKA protocol using the Mixed Strand Space model, revealing vulnerabilities within the protocol. Their study also presented 21 attack scenarios specific to 5G-AKA and highlighted security features not supported by 5G-AKA. Furthermore, ref. [17] identified shortcomings, such as the lack of support for forward secrecy through an analysis of 5G-AKA. In this regard, improved protocols, including [3,16,17,20,21], have been proposed to address such vulnerabilities in 5G-AKA.

**SUCI-AKA**, proposed by [20], aims to achieve forward secrecy for the anchor key $K_{SEAF}$. For this goal, when generating the master session key $K_{AUSF}$, the protocol reuses the shared key $k_{HN}$, which is exchanged through ECIES to encrypt SUPI. In more details, the sequence number SQN is replaced with $k_{HN}$ during the generation of $K_{AUSF}$. Such an approach enhances the security of the anchor and sub-session keys derived from $K_{AUSF}$ while still allowing the UE to verify if the received messages and their message authentication code (MAC) are fresh. However, SUCI-AKA can not support forward secrecy because the anchor key $K_{SEAF}$ is compromised when the long-term key k, shared between the UE and the HN, and the HN’s private key ${\mathrm{sk}}_{HN}$ are leaked.

**5G-IPAKA**, proposed by [17], aims to provide mutual authentication between the UE and the SN, enhanced security for the anchor key and authentication vector (AV), and key confirmation. This protocol tries to provide forward secrecy by applying the ECIES secret $k_{HN}$ to generate the anchor key $K_{SEAF}$, from which sub-sessions keys are then derived. Moreover, it lets the HN send $K_{SEAF}$ to the SN before the UE authentication. In this way, 5G-IPAKA enables the UE to authenticate the SN by verifying the SN’s message authentication code computed with $K_{SEAF}$ in addition to the HN. Similar to this, the UE is authenticated to the SN through its message authentication code. As a result, 5G-IPAKA achieves mutual authentication between the SN and the UE and between the HN and the UE while supporting key confirmation. However, if k and ${\mathrm{sk}}_{HN}$ are leaked, an attacker can reconstruct the anchor key $K_{SEAF}$ for malicious purposes while breaking forward secrecy. Furthermore, active attacks by malicious SNs are also possible since the HN delivers $K_{SEAF}$ to the SN without authenticating the UE. Finally, this protocol leads to compatibility issues with Subscriber Identity Modules (SIMs) by proposing a structure that deviates from the existing standard specification.

**5GAKA-LCCO**, proposed by [21], aims to improve high communication and computation overheads as well as address SUCI replay attacks present in 5G-IPAKA. In this protocol, the SN first creates the random number and timestamp ${\mathrm{RAND}}_{SN}$ and $T_{SN}$, and sends to the UE these values, which are then applied to generate the key block with the long-term shared key k and the ECIES secret $k_{HN}$. Note that the generated key block is not only used to compute the UE’s SUCI but also to authenticate the UE to the HN. Upon a receipt of the new SUCI, the HN decrypts it into the corresponding SUPI, counts on its timestamp $T_{HN}$ to validate the received $T_{SN}$, and authenticates the UE. In such a way, the authentication process is optimized to have one round trip, reducing the computation and communication overhead. Also, the HN utilizes timestamps to prevent the SUCI replay and Denial-of-Service (DoS) attacks on itself while enhancing the security of the session keys by deriving $K_{AUSF}$ from ${\mathrm{sk}}_{HN}$, k, ${\mathrm{RAND}}_{SN}$, and $T_{SN}$. However, it should be noted that when generating $K_{AUSF}$, both k and ${\mathrm{sk}}_{HN}$ are utilized. Therefore, if k and ${\mathrm{sk}}_{HN}$ are leaked, an attacker can recover $K_{AUSF}$ and conduct subsequent malicious attacks. Furthermore, to address SUCI replay attacks, 5GAKA-LCCO introduces freshness to SUCI by utilizing $T_{SN}$. However, this approach requires time synchronization, which may pose challenges in situations such as roaming. Consequently, the use of $T_{SN}$ is not desirable for mobile telecommunication scenarios. Furthermore, 5GAKA-LCCO exhibits an unconventional protocol flow compared to the 5G-AKA standard, and the differences in the AuthenticateSIMcommand can lead to compatibility issues, particularly with Legacy Universal Subscriber Identity Modules (USIMs), potentially resulting in backward compatibility problems.

**5G-AKA**${}^{\prime }$, introduced by [16], focuses on addressing linkability attacks by reusing the ECIES secret $k_{HN}$, which is used to protect SUPI in the initial step. In this protocol, the HN encrypts its randomly generated number RAND into ${\mathrm{RAND}}^{\prime }$ with $k_{HN}$, then sending to the UE the encrypted result instead of RAND along with the authentication token AUTN. At this point, it is worth noting that since the UE trusts the freshness of $k_{HN}$, it also trusts the freshness of ${\mathrm{RAND}}^{\prime }$. Therefore, if successfully decrypting ${\mathrm{RAND}}^{\prime }$ with $k_{HN}$, the UE can trust the freshness of its received AUTN, thereby detecting the message replay attack prior to arriving at the Sync_Failure while defending against the linkability attack. In spite of such a successful defense against the linkability attack, this protocol is vulnerable to active attacks by malicious SNs because it allows the HN to send $K_{SEAF}$ to the SN without authentication to the UE. More importantly, 5G-AKA${}^{\prime }$ fails to achieve forward secrecy because the old anchor keys can be recovered if the long-term key k shared between the UE and the HN is leaked.

## 3. Preliminaries

### 3.1. Notations

Table 1 shows abbreviations and notations to be used throughout this paper.

### 3.2. Elliptic Curve Integrated Encryption Scheme

The Elliptic Curve Integrated Encryption Scheme (ECIES) [24,25] is a well-known hybrid encryption scheme consisting of a Key Encapsulation Mechanism (KEM) and a Data Encapsulation Mechanism (DEM) where messages of arbitrary length can be encrypted. This scheme is a key component of 5G-AKA.

The ECIES-KEM has the following three algorithms:

- KeyGen(pp): On input of a public parameter pp, the algorithm outputs a public-private key pair (PK,sk) such that PK=sk·G, where pp is an elliptic curve parameter standardized in secp256r1 [26], and G∈pp is a base point.
- Encap(PK): On input of a public key PK, the algorithm generates an ephemeral public-private key pair (R,r) such that R=r·G, and then outputs a ciphertext $C_{0}=R$ and a shared key $k_{s}=\mathrm{KDF}\left(r\cdot PK\right)$, where KDF is a key derivation function.
- $\mathrm{Decap}(sk,C_{0})$: On input of a ciphertext $C_{0}$ and a private key sk, the algorithm outputs the shared key $k_{s}=\mathrm{KDF}\left(sk\cdot C_{0}\right)$.

The ECIES-DEM has the following two algorithms:

- $\mathrm{SEnc}(k_{s},M)$: On input of a key $k_{s}$ and a message *M*, the algorithm first parses $k_{s}$ as $k_{1}\left|\right|k_{2}$, computes $C_{1}=\mathrm{ENC}\left(k_{1},M\right)$ and $C_{2}=\mathrm{MAC}\left(k_{2},C_{1}\right)$, and then outputs $\left(C_{1},C_{2}\right)$, where ENC is an encryption part of a symmetric encryption scheme and MAC is a message authentication code.
- $\mathrm{SDec}(k_{s},\left(C_{1},C_{2}\right))$: On input of a ciphertext $\left(C_{1},C_{2}\right)$ and a key $k_{s}$, the algorithm first parses $k_{s}$ as $k_{1}\left|\right|k_{2}$. If $C_{2}\neq \mathrm{MAC}\left(k_{2},C_{1}\right)$, it outputs ⊥. Otherwise, the algorithm outputs $M=\mathrm{DEC}(k_{1},C_{1})$, where DEC is a decryption part of a symmetric encryption scheme.

## 4. A 5G-AKA Protocol for Forward Secrecy

**Figure 2:**
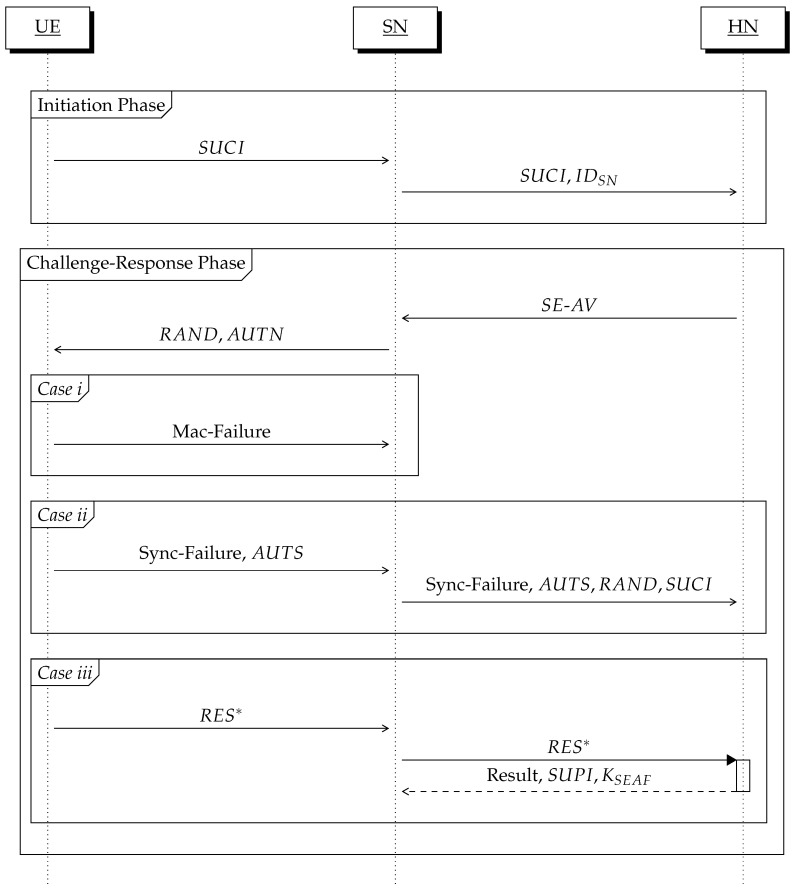
The 5G-AKA-FS protocol.

In this section, we propose a 5G-AKA protocol for forward secrecy (for short, 5G-AKA-FS) that is compatible with the current 3GPP standards [3], and the proposed protocol is shown in Figure 2. Before executing the 5G-AKA-FS protocol, UE holds $(k,\;PK_{HN},\;SUPI,$ $SQN_{UE})$ secretly and HN stores $\left(k,\;sk_{HN},\;ID_{HN},\;SQN_{HN}\right)$ secretly. Also, SN stores $ID_{SN}$. We denote by $H_{\mathrm{SHA}-256}$ the SHA-256 cryptographic hash function.

### 4.1. The Initiation Phase: Step 1

In this phase, UE sends its SUPI as an encrypted form using ECIES [24,25] with HN’s public key $PK_{HN}$. Correspondingly, HN decrypts SUCI with its private key $sk_{HN}$. Upon successful completion of Step 1, we proceed to Step 2 by selecting the 5G-AKA-FS among various methods. Before choosing the authentication method, the Step 1 process unfolds as follows:

#### 4.1.1. Step 1.1 (UE)


**Step 1.1 (UE)**
*Inputs.* With HN’s public key $PK_{HN}$, UE executes the followings:
*The Protocol:*

Generate an ephemeral private-public key pair (r,R) such that R=r·GWith $PK_{HN}$, compute a ciphertext $C_{0}=R$ and a key $k_{UE}=\mathrm{KDF}\left(r\cdot PK_{HN}\right)$Parse the shared key $k_{UE}$ as $k_{1}\left|\right|k_{2}$Compute $C_{1}=\mathrm{ENC}\left(k_{1},SUPI\right)$ and $C_{2}=\mathrm{MAC}(k_{2},$$C_{1})$Set $SUCI\leftarrow (C_{0},C_{1},C_{2})$
*Outputs.* UE sends (SUCI) to SN.

#### 4.1.2. Step 1.2 (SN)


**Step 1.2 (SN)**
*Inputs.* SN receives (SUCI) from UE.*Outputs.* SN sends $\left(SUCI,ID_{SN}\right)$ to HN.

#### 4.1.3. Step 1.3 (HN)


**Step 1.3 (HN)**
*Inputs.* Upon receiving $\left(SUCI,ID_{SN}\right)$ from SN, HN executes the followings:
*The Protocol:*

Compute a shared key $k_{HN}=\mathrm{KDF}\left(sk_{HN}\cdot C_{0}\right)$Parse the shared key $k_{HN}$ as $k_{1}\left|\right|k_{2}$Retrieve the corresponding *k* and $SQN_{HN}$ from its database
*Outputs.* HN outputs ⊥ if $C_{2}\neq \mathrm{MAC}\left(k_{2},C_{1}\right)$. Otherwise, it outputs $SUPI=\mathrm{DEC}(k_{1},C_{1})$.

### 4.2. The Challenge-Response Phase: Step 2

**Figure 3:**
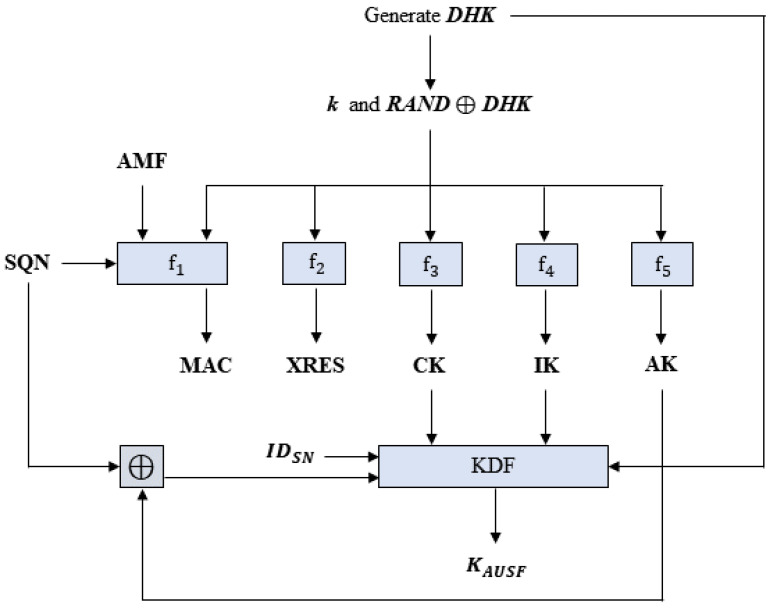
Computations of MAC,XRES,CK,IK,AK, and $K_{AUSF}$ in 5G-AKA-FS.

In this phase, UE and HN authenticate each other via a challenge-response method and establish anchor keys (i.e., $K_{SEAF}$) together with SN. A key idea is that we set a Diffie–Hellman public key *Y* as a random challenge *RAND*, and a Diffie–Hellman key DHK is used as a key material for forward secrecy. This phase uses a series of HMAC-SHA-256 cryptographic key derivation functions $f_{1},f_{2},f_{3},f_{4},f_{5},f_{1}^{*}$, and $f_{5}^{*}$, as specified by TS 33.501 [3] (see also Figure 3).

#### 4.2.1. Step 2.1 (HN)


**Step 2.1 (HN)**
*Inputs.* Using $SQN_{HN}$, *k*, and DHK, HN generates an Authentication Vector $SE-AV=(RAND,AUTN,HXRES^{*})$ as follows:
*The Protocol:*

Generate an ephemeral private-public key pair (y,Y) such that Y=y·GCompute a Diffie–Hellman key $DHK=y\cdot C_{0}$Set RAND←Y as a challenge (The 128-bit randomness of RAND is guaranteed since *y* is randomly chosen from the 128-bit key space. )Compute $MAC\leftarrow f_{1}\left(k,SQN_{HN},AMF,RAND⊕DHK\right)$ andan anonymous key $AK\leftarrow f_{5}\left(k,RAND⊕DHK\right)$Set $AUTN\leftarrow (AK⊕SQN_{HN},AMF,MAC)$Compute $CK\leftarrow f_{3}\left(k,RAND⊕DHK\right)$ and $IK\leftarrow f_{4}\left(k,RAND⊕DHK\right)$Compute expected responses $XRES\leftarrow f_{2}\left(k,RAND⊕DHK\right)$,$XRES^{*}\leftarrow \mathrm{KDF}(CK||IK,ID_{SN}\left||RAND||XRES\right)$, and

$HXRES^{*}\leftarrow \mathrm{LEFT}(128,H_{\mathrm{SHA}-256}(RAND||XRES^{*}))$

Derive $K_{AUSF}\leftarrow \mathrm{KDF}(CK||IK,ID_{SN}||AK⊕SQN_{HN}\left||DHK\right)$ and

$K_{SEAF}\leftarrow \mathrm{KDF}\left(K_{AUSF},ID_{SN}\right)$

Increase $SQN_{HN}$ by 1 (i.e., $SQN_{HN}\leftarrow SQN_{HN}+1$)Set $HE-AV\leftarrow (RAND,AUTN,XRES^{*},K_{AUSF})$ and $SE-AV\leftarrow (RAND,AUTN,HXRES^{*})$
*Outputs.* HN sends (SE-AV) to SN.

#### 4.2.2. Step 2.2 (SN)


**Step 2.2 (SN)**
*Inputs.* SN receives (SE-AV) from HN and stores (RAND, $HXRES^{*})$.*Outputs.* SN sends (RAND,AUTN) to UE.

#### 4.2.3. Step 2.3 (UE)


**Step 2.3 (UE)**
*Inputs.* Upon receiving (RAND,AUTN) from SN, UE executes the following steps:
In the UE, the ME forwards the received RAND and AUTN to the SIMThe SIM computes a Diffie–Hellman key DHK=r·RANDRun the SIM card command AUTHENTICATE(RAND,DHK,AUTN)Compute $AK\leftarrow f_{5}\left(k,RAND⊕DHK\right)$Parse AUTN as (CONC,AMF,MAC)De-conceal $SQN_{HN}\leftarrow AK⊕CONC$Check $f_{1}\left(k,SQN_{HN},AMF,RAND⊕DHK\right)=MAC$
If this check does not pass, the SIM card returns ⊥ and then UE sends a failure message Mac_Failure to SN (see ***Case i***)Otherwise, proceed to the next stepCheck $SQN_{UE}<SQN_{HN}<SQN_{UE}+\Delta$ (The first condition $SQN_{UE}<SQN_{HN}$ ensures the freshness of (RAND,AUTN). Also, the second condition $SQN_{HN}<SQN_{UE}+\Delta$, which is optional in the non-normative Annex C of TS 33.102 [27], prevents a wrap-around of $SQN_{UE}$. For example, if Δ is too small (i.e., Δ=2), an attacker can make a synchronization failure by sending SUCI computed by the attacker with a fake SUPI. After this attack, the honest UE and HN can no longer authenticate each other. In TS 33.102 [27], a recommended value of Δ is $2^{28}$ so as to decrease the synchronization failure rate.)
If this check does not pass, the SIM card computes$MAC^{*}\leftarrow f_{1}^{*}\left(k,SQN_{UE},AMF,RAND⊕DHK\right)$ and returns$AUTS\leftarrow (AK^{*}⊕SQN_{UE},AMF,MAC^{*})$ where $AK^{*}\leftarrow f_{5}^{*}\left(k,RAND⊕DHK\right)$. Then, UE re-synchronizes with HN by sending a failure message Sync_Failure and AUTS to SN (see ***Case ii***)Otherwise, proceed to the next stepSet $SQN_{UE}\leftarrow SQN_{HN}$Compute $CK\leftarrow f_{3}\left(k,RAND⊕DHK\right)$ and $IK\leftarrow f_{4}\left(k,RAND⊕DHK\right)$Compute $RES\leftarrow f_{2}\left(k,RAND⊕DHK\right)\;$ and SIM returns RES, CK, and IK to METhe ME calculates $RES^{*}\leftarrow \mathrm{KDF}(CK||IK,ID_{SN}\left||RAND||RES\right)$ and derives $K_{AUSF}\leftarrow \mathrm{KDF}(CK||IK,ID_{SN}\left||CONC|\right|$DHK) and $K_{SEAF}\leftarrow \mathrm{KDF}\left(K_{AUSF},ID_{SN}\right)$The UE returns $\left(K_{SEAF},RES^{*}\right)$
*Outputs.* The UE stores $K_{SEAF}$ and sends $RES^{*}$ to SN (see ***Case iii***)

*Case i*  **(The SIM card returns** ⊥**):** The UE sends a failure message Mac_Failure to SN.
*Case ii*  **(The SIM card returns** AUTS**):** The UE re-synchronizes with HN by sending a failure message Sync_Failure and AUTS to SN. Upon receiving (Sync_Failure,AUTS), SN sends (Sync_Failure,AUTS,RAND,SUCI) to HN. Then, HN parses AUTS as $\left(AK^{*}⊕SQN_{UE},AMF,MAC^{*}\right)$, and de-conceals $SQN_{UE}$$\leftarrow AK^{*}⊕SQN_{UE}⊕f_{5}^{*}\left(k,RAND⊕DHK\right)$. Next, HN checks its authenticity by comparing $MAC^{*}=f_{1}^{*}\left(k,SQN_{UE},AMF,RAND⊕DHK\right)$. If the check holds, HN re-sets $SQN_{HN}$ by $SQN_{UE}+1$ (i.e., $SQN_{HN}\leftarrow SQN_{UE}+1$).
*Case iii*  **(The SIM card returns** $\left(K_{SEAF},RES^{*}\right)$**):** The UE stores $K_{SEAF}$ and sends $\left(RES^{*}\right)$ to SN. Upon receiving $\left(RES^{*}\right)$, SN computes a hashed value $HRES^{*}\leftarrow \mathrm{LEFT}(128,H_{\mathrm{SHA}-256}(RAND||RES^{*}))$ and checks its validity by comparing $HRES^{*}=HXRES^{*}$. If $HRES^{*}=HXRES^{*}$, SN forwards $\left(RES^{*}\right)$ to HN. Next, HN authenticates UE by comparing $RES^{*}=XRES^{*}$. If $RES^{*}=XRES^{*}$, HN sends its result and $\left(SUPI,K_{SEAF}\right)$ to SN. The SN continues the protocol only if both checks hold, and aborts the protocol otherwise. When all checks pass, UE and SN communicate with session keys derived from anchor keys (i.e., $K_{SEAF}$) in the subsequent 5G procedures. According to TS 33.501 [3], UE and SN should confirm the keys agreed and the identities of each other implicitly through the successful use of keys in subsequent procedures, which can be expressed by a key-confirmation round trip with $K_{SEAF}$.

## 5. Formal Verification

**Figure 4:**
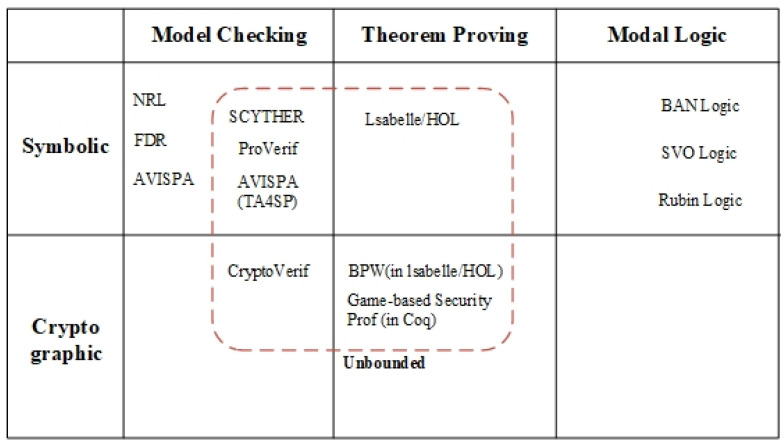
Types of formal verification.

To verify the security of the protocol, various formal verification methods are used as shown in Figure 4. Among several formal verification methods, the proposed protocol is verified through two methods: BAN Logic and ProVerif. BAN Logic is a representative method of Modal Logic and one of the widely used formal verification methods. With its decisiveness on the result, it can be fully trusted once the verification process is correct. However, each verification method has its pros and cons. BAN Logic does not consider dishonest reasoning. In other words, it cannot detect the attack of malicious participants. Thus, for precise verification results, we have included ProVerif as the second verification tool. ISO29129-1, a document for protocol verification framework, guides to formally verify the protocol using the automated prover [28]. ProVerif is one of the state-of-the-art verification tools that meets the guidelines. It can formally verify the protocol in an unbounded session environment and detect malicious attacks on the protocol. By using two complementary verification methods, BAN Logic and ProVerif, we have come up with reliable verification results.

### 5.1. Formal Verification via BAN Logic

The results of BAN Logic are driven by the idealization, assumption, goals, and derivation phase. The notations and rules of BAN Logic used in the above process are shown in Table 2 and Table 3. Excluding messages that are not encrypted, only messages protected by secret keys or secret information between communication participants, such as encryption, digital signature, and message authentication code are expressed. Second, in the Assumption step, preconditions for communication, such as network environment and home, are defined in a form that can be applied in BAN Logic. Third, in the goal step, the security goal to be required in the proposed protocol is defined. Finally, in the derivation step, it shows a series of processes to derive the security attributes defined in the goals through the BAN Logic rule. The results verified through BAN Logic in this paper are as follows.

#### 5.1.1. The Initiation Phase: Step 1

The idealization form of the Initiation Phase of the protocol is shown below:

(1) $$\begin{array}{cc} \; & \mathrm{UE}\to \mathrm{HN}\;:\;{\langle \left\{\mathrm{SUPI},\mathrm{UE}\overset{k_{2}}{⇋}\mathrm{HN}\right\}\rangle }_{k_{1}} \end{array}$$

We added Assumptions (2) to (5) to verify through BAN Logic.

(2) $$\begin{array}{cc} \; & \mathrm{HN}|\equiv \mathrm{UE}\overset{k_{1}}{⇋}\mathrm{HN} \end{array}$$

(3) $$\begin{array}{cc} \; & \mathrm{HN}|\equiv \#(\mathrm{UE}\overset{k_{1}}{⇋}\mathrm{HN}) \end{array}$$

(4) $$\begin{array}{cc} \; & \mathrm{HN}|\equiv \mathrm{UE}\overset{k_{2}}{↔}\mathrm{HN} \end{array}$$

(5) $$\begin{array}{cc} \; & \mathrm{HN}|\equiv \#(\mathrm{UE}\overset{k_{2}}{↔}\mathrm{HN}) \end{array}$$

Technically, all the aforementioned assumptions could be invalidated as the HN inherently lacks trust in the UE’s public key ${\mathrm{PK}}_{UE}$. However, in the case of 5G AKA, since it was designed to tolerate a replay attack or Man-in-the-Middle (MITM) attack on the public key, we followed the standard and added the above assumption to continue this analysis.

Therefore, we set a security goal that HN believes that UE believes in SUPI, and derived this goal through the derivation step.

(6) $$\begin{array}{cc} \; & \mathrm{HN}|\equiv \mathrm{UE}\;|\equiv \mathrm{SUPI} \end{array}$$

from (1), and we derive

(7) $$\begin{array}{cc} \; & \mathrm{HN}◁{\langle {\left\{\mathrm{SUPI},\;\mathrm{UE}\overset{k_{2}}{⇋}\mathrm{HN}\right\}}_{k_{2}},\mathrm{UE}\overset{k_{1}}{⇋}\mathrm{HN}\rangle }_{k_{1}}\;\mathrm{by}\;\left(1\right) \end{array}$$

(8) $$\begin{array}{cc} \; & \mathrm{HN}|\equiv \mathrm{UE}|∼\left({\left\{\mathrm{SUPI},\mathrm{UE}\overset{k_{2}}{⇋}\mathrm{HN}\right\}}_{k_{2}},\;\mathrm{UE}\overset{k_{1}}{⇋}\mathrm{HN}\right)\;\mathrm{by}\;(7),\;(2),\;\mathrm{MM} \end{array}$$

(9) $$\begin{array}{cc} \; & \mathrm{HN}|\equiv \mathrm{UE}|\equiv \left\{\mathrm{SUPI},\;\mathrm{UE}\overset{k_{2}}{⇋}\mathrm{HN}\right\}\;\mathrm{by}\;(8),\;(3),\;\mathrm{FR},\;\mathrm{NV},\;\mathrm{BC} \end{array}$$

(10) $$\begin{array}{cc} \; & \mathrm{HN}|\equiv \mathrm{UE}|\equiv \mathrm{SUPI}\;\mathrm{by}\;(9),\;(4),\;\mathrm{MM},\;(5),\;\mathrm{FR},\;\mathrm{NV},\;\mathrm{BC} \end{array}$$

According to the derivation above, the proposed security protocol can achieve the security goal (6) stated in the goal. This means that the HN believes that UE believes about SUPI, in the initiation phase of the proposed protocol.

#### 5.1.2. The Challenge-Response Phase: Step 2

The idealization form of the Challenge-Response Phase of the protocol is shown below:

(11) $$\begin{array}{cc} \mathrm{HN}\to \mathrm{UE}\;: & \;\overset{Y}{\mapsto }\mathrm{HN},\;{\left\{SQN_{HN}\right\}}_{AK},\;{\langle SQN_{HN},\;\mathrm{AMF},\overset{Y}{\mapsto }\mathrm{HN},\;\mathrm{UE}\overset{x\cdot y\cdot G}{↔}\mathrm{HN},\;\mathrm{UE}\overset{K_{SEAF}}{↔}\mathrm{HN}\;\rangle }_{k} \end{array}$$

(12) $$\begin{array}{cc} \mathrm{UE}\to \mathrm{SN}\;:\; & {\mathrm{RES}}^{*} \end{array}$$

(13) $$\begin{array}{cc} \mathrm{UE}\to \mathrm{HN}\;:\; & {\langle ID_{SN},\;\overset{Y}{\mapsto }\mathrm{HN},\;\mathrm{UE}\overset{x\cdot y\cdot G}{↔}\mathrm{HN},\mathrm{UE}\overset{K_{SEAF}}{↔}\mathrm{SEAF}\rangle }_{k} \end{array}$$

(14) $$\begin{array}{cc} \mathrm{HN}\to \mathrm{SN}\;:\; & \left\{\mathrm{SUPI},\;\mathrm{UE}\overset{K_{SEAF}}{↔}\mathrm{SEAF},\right.{\left.\left(\mathrm{UE}|\equiv \mathrm{UE}\overset{K_{SEAF}}{↔}\mathrm{SEAF}\right)\right\}}_{K_{N12}} \end{array}$$

Unlike HN in (14), in (13), due to no knowledge on k, SN treats ${\mathrm{RES}}^{*}$ as an unrecognized simple value. We added assumptions from (15)–(26) to derive the security goal.

(15) $$\begin{array}{cc} \; & \mathrm{UE}|\equiv \mathrm{UE}\overset{K}{↔}\mathrm{HN} \end{array}$$

(16) $$\begin{array}{cc} \; & \mathrm{UE}|\equiv \;\overset{X}{\mapsto }\mathrm{UE} \end{array}$$

(17) $$\begin{array}{cc} \; & \mathrm{UE}|\equiv \#(SQN_{HN}) \end{array}$$

(18) $$\begin{array}{cc} \; & \mathrm{UE}|\equiv \#(K_{SEAF}) \end{array}$$

(19) $$\begin{array}{cc} \; & \mathrm{SN}|\equiv \;\overset{Y}{\mapsto }\mathrm{HN} \end{array}$$

(20) $$\begin{array}{cc} \; & \mathrm{SN}|\equiv H\left({RES}^{*},\;\overset{Y}{\mapsto }HN\right) \end{array}$$

(21) $$\begin{array}{cc} \; & \mathrm{HN}|\equiv \mathrm{UE}\overset{K}{⇋}\mathrm{HN} \end{array}$$

(22) $$\begin{array}{cc} \; & \mathrm{HN}|\equiv \#(\;\overset{Y}{\mapsto }\mathrm{HN}) \end{array}$$

(23) $$\begin{array}{cc} \; & \mathrm{SN}|\equiv \mathrm{SN}\overset{K_{N12}}{↔}\mathrm{HN} \end{array}$$

(24) $$\begin{array}{cc} \; & \mathrm{HN}|\equiv \#(\mathrm{SN}\overset{K_{N12}}{↔}\;\mathrm{HN}) \end{array}$$

(25) $$\begin{array}{cc} \; & \mathrm{SN}|\equiv \mathrm{HN}\Rightarrow \mathrm{UE}\overset{K_{SEAF}}{↔}\mathrm{SEAF} \end{array}$$

(26) $$\begin{array}{cc} \; & \mathrm{SN}|\equiv \mathrm{HN}\Rightarrow \left(\mathrm{UE}|\equiv \mathrm{UE}\overset{K_{SEAF}}{↔}\mathrm{SEAF}\right) \end{array}$$

$K_{SEAF}$ is derived based on the ECIES ephemeral key pair performed once again in HN, and the UE can also derive $K_{SEAF}$ through the ECIES process. Therefore, since the UE can trust that $K_{SEAF}$ is fresh, (18) is added. Also, (19) and (20) are added because SN receives $\mathrm{RAND}=\;\overset{Y}{\mapsto }\mathrm{HN}$ and ${\mathrm{HXRES}}^{*}$ from HN via secure channel and counts on HN.

(27) $$\begin{array}{cc} \; & \mathrm{UE}|\equiv \mathrm{HN}|\equiv \;\overset{Y}{\mapsto }\mathrm{HN} \end{array}$$

(28) $$\begin{array}{cc} \; & \mathrm{UE}|\equiv \mathrm{UE}\overset{x\cdot y\cdot G}{↔}\mathrm{HN} \end{array}$$

(29) $$\begin{array}{cc} \; & \mathrm{UE}|\equiv \mathrm{HN}|\equiv \;\mathrm{UE}\overset{x\cdot y\cdot G}{↔}\mathrm{HN} \end{array}$$

(30) $$\begin{array}{cc} \; & \mathrm{UE}|\equiv \mathrm{UE}\overset{K_{SEAF}}{↔}\mathrm{HN} \end{array}$$

(31) $$\begin{array}{cc} \; & \mathrm{UE}|\equiv \mathrm{HN}|\equiv \;\mathrm{UE}\overset{K_{SEAF}}{↔}\mathrm{SEAF} \end{array}$$

(32) $$\begin{array}{cc} \; & \mathrm{HN}|\equiv \mathrm{UE}|\equiv \mathrm{UE}\overset{x\cdot y\cdot G}{↔}\mathrm{HN} \end{array}$$

(33) $$\begin{array}{cc} \; & \mathrm{HN}|\equiv \mathrm{UE}|\equiv \mathrm{UE}\overset{K_{SEAF}}{↔}\mathrm{HN} \end{array}$$

(34) $$\begin{array}{cc} \; & \mathrm{SN}|\equiv \mathrm{HN}|\equiv \mathrm{UE}\overset{K_{SEAF}}{↔}\mathrm{SEAF} \end{array}$$

(35) $$\begin{array}{cc} \; & \mathrm{SN}|\equiv \mathrm{UE}|\equiv \mathrm{UE}\overset{K_{SEAF}}{↔}\mathrm{SEAF} \end{array}$$

from (11), we derive

(36) $$\begin{array}{cc} \; & \mathrm{UE}◁\;\overset{Y}{\mapsto }\mathrm{HN}\;\mathrm{by}\;\left(11\right) \end{array}$$

Here, given the ephemeral ECIES public key of HN, UE derives DHK=x·Y=x·y·G and then computes $AK=f_{5}\left(k,Y⨁\left(x\cdot y\cdot G\right)\right)$. At this point, despite no trust in Y, UE can be sure that AK is a good key shared between HN and itself. This is because AK is derived by inputting k and x where k is only known to both UE and HN and x is its own fresh ephemeral ECIES private key. As a result, UE gains the belief (37) as follows.

(37) $$\begin{array}{cc} \; & \mathrm{UE}|\equiv \mathrm{UE}\overset{AK}{↔}\mathrm{HN}\;\mathrm{by}\;(36),\;(15),\;\left(16\right) \end{array}$$

(38) $$\begin{array}{cc} \; & \mathrm{UE}◁{\left\{SQN_{HN}\right\}}_{AK}\;\mathrm{by}\;\left(11\right) \end{array}$$

(39) $$\begin{array}{cc} \; & \mathrm{UE}|\equiv \mathrm{HN}|\equiv SQN_{HN}\;\mathrm{by}\;(38),\;(37),\;\mathrm{MM},\;(20),\;\mathrm{NV} \end{array}$$

Note that after recovery, ${\mathrm{SQN}}_{HN}$ is checked for its freshness. For this procedure, we add the assumption (17). Now, UE possesses the valid ${\mathrm{SQN}}_{HN}$.

(40) $$\begin{array}{cc} \; & \mathrm{UE}◁{\langle SQN_{HN},\;AMF,\;\overset{Y}{\mapsto }\mathrm{HN},\;\mathrm{UE}\overset{K_{SEAF}}{↔}\mathrm{SEAF}\rangle }_{k}\;\mathrm{by}\;\left(11\right) \end{array}$$

(41) $$\begin{array}{cc} \; & \mathrm{UE}◁\mathrm{HN}|∼\left(SQN_{HN},\;AMF,\;\overset{Y}{\mapsto }\mathrm{HN},\right.\left.\mathrm{UE}\overset{K_{SEAF}}{↔}\mathrm{SEAF}\right)\;\mathrm{by}\;(40),\;(15),\;\mathrm{MM} \end{array}$$

(42) $$\begin{array}{cc} \; & \mathrm{UE}|\equiv \mathrm{HN}|\equiv \left(SQN_{HN},\;AMF,\;\overset{Y}{\mapsto }\mathrm{HN},\right.\left.\mathrm{UE}\overset{K_{SEAF}}{↔}\mathrm{SEAF}\right)\;\mathrm{by}\;(41),\;(18),\;\mathrm{FR},\;\mathrm{NV} \end{array}$$

(43) $$\begin{array}{cc} \; & \mathrm{UE}|\equiv \mathrm{HN}|\equiv \;\overset{Y}{\mapsto }\mathrm{HN}\;\mathrm{by}\;\left(11\right) \end{array}$$

Even if *RAND* and *AUTN* are replayed, a linkability attack does not occur in the UE. Because freshness is verified based on the key derived through ECIES rather than ${\mathrm{SQN}}_{HN}$ during MAC check, when a replay attack occurs, the Sync_Failure step is not performed, and MAC_Failure is executed to stop authentication, so unlinkability is supported.

In addition, UE obtains the indirect belief on $\overset{Y}{\mapsto }$HN, which helps this protocol to defend against the Man-in-The-Middle attacks. Based on (43), UE proceeds to gain the direct belief on $\mathrm{UE}\overset{x\cdot y\cdot G}{↔}\mathrm{HN}$ as follows.

(44) $$\begin{array}{cc} \; & \mathrm{UE}|\equiv \mathrm{UE}\overset{x\cdot y\cdot G}{↔}\mathrm{HN}\;\mathrm{by}\;(43),\;(16),\;\mathrm{DH} \end{array}$$

(45) $$\begin{array}{cc} \; & \mathrm{UE}|\equiv \mathrm{HN}|\equiv \;\mathrm{UE}\overset{x\cdot y\cdot G}{↔}\mathrm{HN}\;\mathrm{by}\;(42),\;\mathrm{BC} \end{array}$$

UE derives $K_{SEAF}$ with the values k, ${\mathrm{SQN}}_{HN}$, Y, x·y·G, ${\mathrm{ID}}_{SN}$. In particular, based on (15), (17), (43), and (44), UE can arrive at the following belief.

(46) $$\begin{array}{cc} \; & \mathrm{UE}|\equiv \mathrm{UE}\overset{K_{SEAF}}{↔}\mathrm{HN}\;\mathrm{by}\;(25),\;(18),\;(43),\;\left(44\right) \end{array}$$

(47) $$\begin{array}{cc} \; & \mathrm{UE}|\equiv \mathrm{HN}|\equiv \;\mathrm{UE}\overset{K_{SEAF}}{↔}\mathrm{SEAF}\;\mathrm{by}\;(42),\;\mathrm{BC} \end{array}$$

At this point, it is confirmed from (44) to (47) that HN is authenticated to UE.

from (12), and we derive

(48) $$\begin{array}{cc} \; & \mathrm{SN}◁{\mathrm{RES}}^{*}\;\;\mathrm{by}\;\left(12\right) \end{array}$$

(49) $$\begin{array}{cc} \; & \mathrm{SN}|\equiv {\mathrm{RES}}^{*}\;\mathrm{by}\;(48),\;(18),\;(19),\;\mathrm{HR} \end{array}$$

Based on (49), SN proceeds this protocol by forwarding ${\mathrm{RES}}^{*}$ to HN.

from (13), and we derive

(50) $$\begin{array}{cccc} & & \mathrm{HN}◁{\langle {\mathrm{ID}}_{SN},\;\overset{Y}{\mapsto }\mathrm{HN},\;\mathrm{UE}\overset{x\cdot y\cdot G}{↔}\mathrm{HN},\;\mathrm{UE}\overset{K_{SEAF}}{↔}\mathrm{HN}\rangle }_{k}\;\;\;\;\mathrm{by}\;\left(13\right) & \end{array}$$

(51) $$\begin{array}{c} \mathrm{HN}|\equiv \mathrm{UE}|\equiv \left({\mathrm{ID}}_{SN},\;\overset{Y}{\mapsto }\mathrm{HN},\;\mathrm{UE}\overset{x\cdot y\cdot G}{↔}\mathrm{HN},\right.\left.\mathrm{UE}\overset{K_{SEAF}}{↔}\mathrm{HN}\right)\\ \mathrm{by}\;(50),\;(21),\;\mathrm{MM},\;(22),\;\mathrm{FR},\;\mathrm{NV} \end{array}$$

(52) $$\begin{array}{ccccc} \; & \; & \mathrm{HN}|\equiv \mathrm{UE}|\equiv \mathrm{UE}\overset{x\cdot y\cdot G}{↔}\mathrm{HN}\;\mathrm{by}\;(51),\;\mathrm{BC} & \; & \end{array}$$

(53) $$\begin{array}{ccccc} \; & \; & \mathrm{HN}|\equiv \mathrm{UE}|\equiv \mathrm{UE}\overset{K_{SEAF}}{↔}\mathrm{HN}\;\mathrm{by}\;(51),\;\mathrm{BC} & \; & \end{array}$$

Based on (51), UE and HN mutually authenticate

from (14), and we derive

(54) $$\begin{array}{cc} \; & \mathrm{SN}◁{\left\{\mathrm{SUPI},\mathrm{UE}\overset{K_{SEAF}}{↔}\mathrm{HN},\left(\mathrm{UE}\overset{K_{SEAF}}{↔}\mathrm{SEAF}\right)\right\}}_{K_{N12}}\;\mathrm{by}\;\left(14\right) \end{array}$$

(55) $$\begin{array}{c} \mathrm{SN}|\equiv \mathrm{HN}|\equiv \left(\mathrm{SUPI},\mathrm{UE}\overset{K_{SEAF}}{↔}\mathrm{HN},\right.\left.\left(\mathrm{UE}|\equiv \mathrm{UE}\overset{K_{SEAF}}{↔}\mathrm{SEAF}\right)\right)\\ \mathrm{by}\;(54),\;(23),\;\mathrm{MM},\;(24),\;\mathrm{FR},\;\mathrm{NV}\; \end{array}$$

(56) $$\begin{array}{cc} \; & \mathrm{SN}|\equiv \mathrm{HN}|\equiv \mathrm{UE}\overset{K_{SEAF}}{↔}\mathrm{SEAF}\;\mathrm{by}\;(55),\;\mathrm{BC} \end{array}$$

(57) $$\begin{array}{cc} \; & \mathrm{SN}|\equiv \mathrm{UE}\overset{K_{SEAF}}{↔}\mathrm{SEAF}\;\mathrm{by}\;(56),\;(25),\;\mathrm{JR} \end{array}$$

(58) $$\begin{array}{cc} \; & \mathrm{SN}|\equiv \mathrm{HN}|\equiv \left(\mathrm{UE}|\equiv \mathrm{UE}\overset{K_{SEAF}}{↔}\mathrm{SEAF}\right)\;\mathrm{by}\;(55),\;\mathrm{BC} \end{array}$$

(59) $$\begin{array}{cc} \; & \mathrm{SN}|\equiv \mathrm{UE}|\equiv \mathrm{UE}\overset{K_{SEAF}}{↔}\mathrm{SEAF}\;\mathrm{by}\;(58),\;(26),\;\mathrm{JR} \end{array}$$

### 5.2. Formal Verification via ProVerif

#### 5.2.1. Implementations and designs

In this section, we formally verify the 5G-AKA-FS protocol using a well-known formal verification tool, ProVerif [23]. We designed the processes of User Equipment (UE), Serving Network (SN), and Home Network (HN) respectively at **UE**, **SN** and **HN** in ProVerif to verify its security properties. Our implementation also contains the process protocol that concludes the proof.

We implemented the processes under Dolev–Yao’s attacker model, where the attacker can access the encrypted data only if it has the correct key to decrypt them. We largely adopt the settings that the implementation of Damir et al. [29] is based on. The differences between ours and Damir et al. are as follows:

1. First, we newly define the symbolic process DHkey for the perfect forward secrecy, that is the Diffie–Hellman Key exchange protocol, in the implementation.
2. Although our settings are similar to Damir et al. [29], the actual internal processes are different as the internal processes of UE, SN, and HN are fairly different from those of Damir et al.’s protocol. We implement those differences in our implementation.

Under Dolev–Yao’s attacker model, we simplify some cryptographic primitives as symbolic functions. Particularly, the Diffie–Hellman key exchange protocol can be implemented as a symbolic function as follows:
**DH Key Exchange protocol**type pubKey.type secKey.⋯fun pk(secKey):pubKey.fun DHkey(secKey,pubKey):bitstring.equation forall sk1:secKey, sk2:secKey;DHKey(sk2,pk(sk1))=DHKey(sk1,pk(sk2)).

For the symmetric key encryption/decryption, we utilized a symbolic function defined in [29] to implement the protocols in our 5G-AKA-FS. Symmetric key encryption/decryption means that a message m is encrypted by senc using a secret key n. Then, the encrypted message senc(m,n) only can be decrypted using sdec with the same key. It is defined as follows:
**Encryption/Decryption**fun senc(bitstring,bitstring):bitstring.reduc forall m:bitstring,n:bitstring;sdec(senc(m,n),n)=m.

#### 5.2.2. Protocol Process

We processed our protocol for the verification. We define the public key of HN using its corresponding private key, skHN, and the public key is broadcasted to all public channels as it is a public parameter. We composited infinite replication of UE, SN and HN processes in parallel for protocol processes.
**Protocol Process**processnew skHN :secKey;new uk :bitstring;new idHN :bitstring;new SNname :bitstring;new SQNUE :bitstring;new delta :bitstring;new SUPI :bitstring;let pkHN = pk(skHN) in out(usch1, (pkHN));out(usch2, (pkHN));out(usch3, (pkHN));(!UE(SUPI,idHN,pkHN,uk,SNname,SQNUE,delta)|!SN(SNname)|!HN(skHN,idHN))

#### 5.2.3. Assertions

Using ProVerif, we can verify if the adversary can access the security parameters by reaching the state where those parameters are available. In our protocol, the private key of HN (skHN), a Long-term key at UE/HN (uk), and the Long-term identity (SUPI) were queried to verify their secrecy.
**Secrecy of Parameters**query attacker(skHN).query attacker(uk).query attacker(SUPI).

The sequence of the protocol can also be verified by modeling how the messages are processed and exchanged among UE, SN and HN. Those can be verified using assertions consisting of events that represent message processing and delivery. We utilize the same naming rules for events with [29]. Therefore, for a query *x*, we formally define events as follows:

- *event UESendReqSN(·):* UE sends a connection request with the required parameters to SN in Step 1.1.
- *event SNRecReqUE(·):* SN receives a connection request with the required parameters from UE in Step 1.2.
- *event SNSendReqHN(·):* SN sends a connection request with the required parameters to HN in Step 1.2.
- *event HNRecReqSN(·):* HN receives the result of a connection request with the other relevant parameters from SN in Step 1.3.
- *event HNSendResSN(·):* HN sends the result of a connection request with the other relevant parameters to SN in Step 2.1.
- *event SNRecResHN(·):* SN receives the result of a connection request with the other relevant parameters from HN in Step 2.2.
- *event SNSendResUE(·):* SN sends the result of a connection request with the other relevant parameters to HN in Step 2.2.
- *event UERecResSN(·):* UE receives the result of a connection request with the other relevant parameters from SN in Step 2.3.
- *event UESendConSN(·):* UE sends authentication parameters to SN in *Case iii* in Step 2.3.
- *event SNSendConHN(·):* SN forwards ($RES^{*}$) to HN in *Case iii* in Step 2.3.
- *event SNRecConUE(·):* SN receives ($RES^{*}$) from UE *Case iii* in Step 2.3.
- *event HNRecConSN(·):* HN receives ($RES^{*}$) from SN *Case iii* in Step 2.3.
- *event HNSendSUPISN(·):* HN sends ($SUPI,K_{SEAF}$) to SN in *Case iii* in Step 2.3.
- *event SNRecSUPIHN(·):* SN receives ($SUPI,K_{SEAF}$) from HN in *Case iii* in Step 2.3.

Using the events described above, we can formally verify the hypothesis “event A ==> event B”, which means that, without the execution of event B, event A cannot be executed. For example, the first two lines of the following ProVerif code of Process Verification imply that SN sends a connection request with the required parameters to HN only if HN receives the result of a connection request with the other relevant parameters from SN.

The following are the all events we verified in the implementation.
**Process Verification**query a:bitstring,b:bitstring;event(HNRecReqSN(a))  ==>   event(SNSendReqHN(b)).query a:bitstring,b:bitstring;event(SNRecResHN(a))  ==>   event(HNSendResSN(b)).query a:bitstring,b:bitstring;event(UERecResSN(a))   ==>   event(SNSendResUE(b)).query a:bitstring,b:bitstring;event(SNRecConUE(a))   ==>  event(UESendConSN(b)).query a:bitstring,b:bitstring;event(HNRecConSN(a))   ==>   event(SNSendConHN(b)).query a:bitstring,b:bitstring;event(SNRecSUPIHN(a))   ==>   event(HNSendSUPISN(b)).

#### 5.2.4. Verification Results

We execute our ProVerif code on the ProVerif online demo website. As a result of the verification, we can conclude that the attacker cannot access the security keys in any state of the process. Moreover, all the sequences of executions of UE, SN, and HN are verified as defined in the protocol.

The summary of the verification results is as follows:
**Verification Summary**Query not attacker(skHN[]) is true.Query not attacker(uk[]) is true.Query not attacker(SUPI[]) is true.Query event(HNRecReqSN(a))  ==>   event(SNSendReqHN(b)) is true.Query event(SNRecResHN(a))  ==>   event(HNSendResSN(b)) is true.Query event(UERecResSN(a))  ==>   event(SNSendResUE(b)) is true.Query event(SNRecConUE(a))  ==>   event(UESendConSN(b)) is true.Query event(HNRecConSN(a))  ==>   event(SNSendConHN(b)) is true.Query event(SNRecSUPIHN(a))  ==>   event(HNSendSUPISN(b)) is true.

## 6. Comparative Analysis

In this section, we conduct a comparative assessment aimed at evaluating the effectiveness of the proposed protocol by considering security requirements as well as the computational and communication overheads. For a starter, six protocols are compared against six security requirements to assess the degree of security they each offer. This analysis showcases that the proposed protocol delivers robust security measures in comparison to the others.

Next, we proceed to determine the computational overhead linked to each protocol. This involves scrutinizing the amount of cryptographic operations required within each security protocol and quantifying the computational overhead through Python. We also evaluate the communication overhead by closely examining the message dimensions described in the 3GPP standard document [3]. For that, the transmitted messages are not only inspected, but also their bit sizes are calculated to measure the communication overhead caused by each protocol.

### 6.1. Security Analysis

The proposed security protocol is compared to the existing protocols based on six security requirements: 5G Network and UE’s Mutual Authentication (AUTH), Secure Key Exchange (SKE), Legacy USIM Compatibility Support (LUCS), LinkaBility Attack (LBA), Active attack by Malicious SN (AMS), and Forward Secrecy for $K_{SEAF}$ (FS). The security requirements that each protocol satisfies are shown in Table 4.

According to the table, all protocols satisfy the requirements for AUTH as well as SKE. However, LUCS is not supported by 5G-IPAKA and 5GAKA-LCCO. 5G-IPAKA introduces a different structure, which may result in compatibility issues. Similarly, 5GAKA-LCCO deviates from the standard by utilizing $T_{SN}$ and follows an unconventional protocol flow, potentially leading to compatibility problems with previous versions, i.e., reverse compatibility problems.

LBA is an attack related to compromising a UE’s location privacy. 5GAKA-LCCO eliminates the process of synchronization failure by reducing round-trip, thus preventing LBA. In the case of 5G-AKA${}^{\prime }$, $k_{HN}$ is reused instead of SQN, which enables it to confirm the freshness of MAC and address LBA. On the other hand, 5G-AKA-FS can defend against LBA because it computes DHK in HN and reflects this key when generating values required for authentication.

In 5G-IPAKA and 5G-AKA${}^{\prime }$, active attacks by malicious SN are possible because the HN delivers $K_{SEAF}$ to the SN without authentication of the UE. 5G-AKA, SUCI-AKA, and 5G-AKA${}^{\prime }$ derive the anchor key $K_{SEAF}$ through the long-term key k, so FS is not supported when k is leaked. 5G-IPAKA and 5GAKA-LCCO used not only k but also HN’s private key ${\mathrm{sk}}_{HN}$ to support FS. However, if ${\mathrm{sk}}_{HN}$ is compromised, FS for $K_{SEAF}$ in the past is not achieved.

### 6.2. Overhead Analysis

To compare the trade-off between security and resource consumption, we have compared the proposed protocol computation and communication overhead. SUCI-AKA, 5G-IPAKA, 5GAKA-LCCO, and 5G-AKA′ are other protocols improvised based on 5G-AKA. However, they do not provide complete FS. EAP-TLS1.3 and EAP-AKA′-FS are both representative protocols on the mobile network field and provide complete FS. The test results provide respectful data for a trade-off between security and resource consumption.

#### 6.2.1. Computation Overhead

Table 5 summarizes the environment used in the experiment. The computation overhead for each protocol was measured by conducting 5000 test runs using the cryptography library in Python 3.10.11.

**Figure 5:**
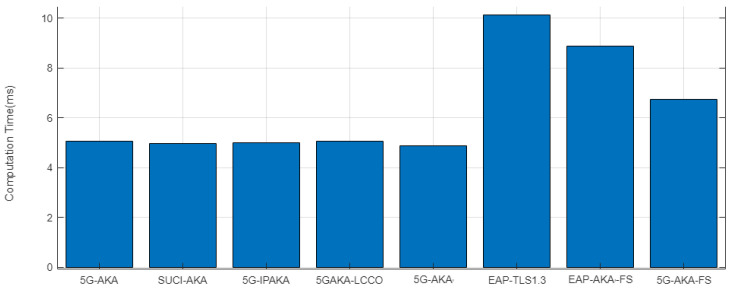
Total Computation Overhead.

As can be seen in a test result shown in Figure 5, 5G-AKA-related protocols have minor differences with 5G-AKA computation overhead. However, these protocols do not completely provide FS. The proposed protocol, with a computation overhead of 6.75 ms, provides FS and has resistance against LBA and AMS. Moreover, as can be seen in comparison with EAP-TLS1.3 and EAP-AKA′-FS, the increase in computation overhead for providing FS in the proposed protocol is low.

The majority of the computation overhead is incurred by ECDH (Elliptic Curve Diffie–Hellman) and digital signature. To provide FS, the protocol must generate a fresh key every session, which 5G-AKA does not do on the HN side, so an increase in overhead to provide FS on protocols is mostly caused by adding fresh ECDH in the key generation phase. In EAP-AKA′-FS, HN and UE generate fresh ECDH keys in the challenge-response phase. However, the proposed protocol, with the reuse of the fresh ECDH key generated in the initiation phase. In Step 1.1, the proposed protocol computation overhead is optimized. Moreover, unlike EAP-TLS1.3, by a succession of 5G-AKA architecture, the proposed protocol does not require a digital signature. These are the reasons the proposed protocol has lower computation overhead than EAP-TLS1.3 and EAP-AKA′-FS. Considering that our proposed protocol provides strong security, this level of computational overhead is deemed acceptable. It represents a trade-off that we are willing to accept to prioritize robust security.

#### 6.2.2. Communication Overhead

Communication overhead refers to the total message size that needs to be transferred in the network for a specific purpose. Based on the 3GPP 33.501 specification, we have analyzed the total size of the messages that are exchanged through the primary authentication phase. Table 6 refers to the size of the messages that consist of the 5G primary authentication phase.

The combination of upper messages concludes the total communication overhead of the 5G-AKA. Table 7 gives the total communication overhead of 5G-AKA, improved protocols, and for practical comparison EAP-TLS1.3 and EAP-AKA′-FS.

For one protocol to offer additional security properties and inherit the structure of 5G-AKA, it is most likely to use additional messages to fulfill that purpose. As a result, 5G-AKA-FS leads to a higher communication overhead than 5G-AKA. However, as compensation for this sacrifice, i.e., additional overhead, the proposed protocol offers forward secrecy by newly generating the HN’s ephemeral ECDH public key and using it as RAND in the initialization phase. Note that in 5G-AKA RAND is a 128-bit random challenge, but in 5G-AKA-FS the RAND challenge is replaced with the HN’s ephemeral ECDH public key, which is 256-bit. This results in 5G-AKA-FS having 384-bit higher communication overhead than 5G-AKA. With only 384-bit extra messages traveling through the network, 5G-AKA-FS achieves resistance against LBA and AMS and provides FS. Among 5G-AKA-related protocols, 5G-AKA-FS is the only improvised protocol that offers all three security properties. Furthermore, despite its increase, the overhead of 5G-AKA-FS remains lower than that of the EAP-TLS-1.3 protocol and EAP-AKA′-FS. The analysis results indicate that the addition of 384 extra bits for three crucial security properties are reasonably justifiable trade-offs.

## 7. Further Discussions

The proposed protocol has both advantages on security properties and overheads. However, implementing 5G-AKA-FS presents another challenge. While it is USIM compatible and does not require hardware exchange, software updates are required on both the UE and the 5G Core. Given that the 5G network serves as the infrastructure for connecting a massive number of devices, testing, and simulation are essential before the updates.

As for security properties, the proposed 5G-AKA-FS protocol has several limitations. For example, our protocol does not provide resistance to key compromise impersonation attacks since an attacker who obtains a permanent key *k* from HN can easily impersonate UE. Also, the proposed protocol does not guarantee security against ephemeral key leakage (e.g., due to side-channel attacks) because the exposure of *r* breaks UE anonymity. Additionally, the security of the proposed protocol relies on the safety of the cryptographic key exchange function ECDH. However, the emergence of quantum computing poses a threat to the security of legacy cryptographic algorithms including ECDH. Therefore, future research on quantum-resistant AKA using Post-Quantum Cryptography is required.

## 8. Conclusions

In this paper, we proposed an improved 5G-AKA (5G-AKA-FS) protocol that provides UE unlinkability and forward secrecy, and is compatible with the 3GPP standard. Implementing the proposed protocol will require the update on UE and 5G Core. However, since it is compatible with the original 5G-AKA it only needs minor software updates. Also, we proved that the 5G-AKA-FS protocol is valid by using formal verification tools BAN Logic and ProVerif. Moreover, we compared the security properties and computation/communication overheads of our 5G-AKA-FS to those of the other existing protocols, including 5G-AKA, SUCI-AKA, 5G-AKA′, 5G-IPAKA, 5GAKA-LCCO, EAP-TLS 1.3, and EAP-AKA′-FS. This comparative analysis demonstrates that the proposed protocol effectively maintains a balance between security and efficiency.

## Figures and Tables

**Table 1:** Abbreviations and notations.

	Meanings
HN	Home Network
UE (ME, SIM)	User Equipment (Mobile Equipment, Subscriber Identity Modules)
SN	Serving Network
SUPI	SUbscriber Permanent Identifier
SUCI	SUbscriber Concealed Identifier
KEM	Key Encapsulation Mechanism
DEM	Data Encapsulation Mechanism
AMF	Access Management Function
AUSF	Authentication Server Function
SEAF	SEcurity Anchor Function
*k*	A permanent key shared between UE and HN
$K_{AUSF}$	A master session key derived from 5G-AKA-FS
$K_{SEAF}$	An anchor key derived from 5G-AKA-FS
$k_{UE}$	UE’s shared key established by ECIES-KEM
$k_{HN}$	HN’s shared key established by ECIES-KEM
$\left(PK_{HN},sk_{HN}\right)$	HN’s ECIES public-private key pair where $PK_{HN}=sk_{HN}\cdot G$
$ID_{SN}$	Unique identifier of SN
$ID_{HN}$	Unique identifier of HN
$SQN_{UE}$	UE’s sequence number
$SQN_{HN}$	HN’s sequence number
RAND	HN’s challenge message
AUTH	Mutual AUTHentication
SKE	Secure Key Exchange
LUCS	Legacy USIM Compatibility Support
LBA	LinkaBility Attack
AMS	Active attack by Malicious SN
FS	Forward Secrecy

**Table 2:** Notations of BAN Logic.

Notation	Meaning
P∣≡X	*P* believes the message *X*
P◁X	*P* receives the message *X*
P∣∼X	*P* previously sent the message *X*
P⇒X	*P* has authority over *X*
#(X)	The message *X* is fresh
${\langle X\rangle }_{K}$	*X* is combined with a secret *K*
${\left\{X\right\}}_{K}$	*X* is encrypted with a key *K*
$P\overset{K}{↔}Q$	*K* is a secret key shared between *P* and *Q*
$\overset{K}{\mapsto }P$	*K* is the public key of *P*
$P\overset{K}{⇋}Q$	*K* is a shared secret between *P* and *Q*

**Table 3:** Rules of BAN Logic.

Rule	Formula
Message Meaning Rule (MM)	$\frac{P\;|\equiv \;P\overset{K}{↔}Q,\;P\;◁\;{\left\{X\right\}}_{K}}{P\;|\equiv \;Q\;|∼\;X}$ $\frac{P\;|\equiv \;P\overset{K}{⇋}Q,\;P\;◁\;{\langle X\rangle }_{K}}{P\;|\equiv \;Q\;|∼\;X}$ $\frac{P\;|\equiv \;\overset{K}{\mapsto }Q,\;P\;◁\;{\left\{X\right\}}_{Q^{-1}}}{P\;|\equiv \;Q\;|∼\;X}$
Nonce Verification Rule (NV)	$\frac{P\;|\equiv \;\#(X),\;P\;|\equiv \;Q\;|∼\;X}{P\;|\equiv Q\;|\equiv \;X}$
Jurisdiction Rule (JR)	$\frac{P\;|\equiv \;Q\;\Rightarrow \;X,\;P\;|\equiv \;Q\;|∼\;X}{P\;|\equiv \;X}$
Freshness Rule (FR)	$\frac{P\;|\equiv \;\#(X)}{P\;|\equiv \;\#(X,\;Y)}$
Decomposition Rule (DR)	$\frac{P\;◁\;(X,\;Y)}{P\;◁\;X}$
Belief Conjunction Rule (BC)	$\frac{P\;|\equiv \;X,\;P\;|\equiv \;Y}{P\;|\equiv \;(X,\;Y)}$ $\frac{P\;|\equiv \;Q\;|\equiv \;(X,\;Y)}{P\;|\equiv \;Q\;|\equiv \;X}$ $\frac{P\;|\equiv \;Q\;|∼\;(X,\;Y)}{P\;|\equiv \;Q\;|∼\;X}$
Hash Rule (HR)	$\frac{P\;|\equiv \;Q\;|∼\;H(X),P\;◁\;X}{P\;|\equiv \;Q\;|∼\;X}$
Diffie–Hellman Rule	$\frac{P\;|\equiv \;Q\;|∼\;\overset{g^{Y}}{\mapsto }\;Q,\;P\;|\equiv \;\overset{g^{X}}{\mapsto }\;P}{P\;|\equiv \;P\;\overset{g^{XY}}{↔}\;Q}$ $\frac{P\;|\equiv \;Q\;|∼\;\overset{g^{Y}}{\mapsto }\;Q,\;P\;|\equiv \;\overset{g^{X}}{\mapsto }\;P}{P\;|\equiv \;P\;\overset{g^{XY}}{⇋}\;Q}$

**Table 4:** Comparison in terms of security requirements with existing protocols.

Protocol	Security Requirements					
	**AUTH**	**SKE**	**LUCS**	**LBA**	**AMS**	**FS**
5G-AKA [3]	◯	◯	◯	×	◯	×
SUCI-AKA [20]	◯	◯	◯	×	◯	×
5G-IPAKA [17]	◯	◯	×	×	×	×
5GAKA-LCCO [21]	◯	◯	×	◯	◯	×
5G-AKA${}^{\prime }$ [16]	◯	◯	◯	◯	×	×
EAP-TLS1.3	◯	◯	×	◯	◯	◯
EAP-AKA${}^{\prime }$-FS	◯	◯	◯	◯	◯	◯
5G-AKA-FS	◯	◯	◯	◯	◯	◯

◯: Support; ×: Not support;

**Table 5:** Experimental environments.

Operating System	Windows 11
CPU	12th Gen Intel(R) Core(TM) i7-12700KF
GPU	NVIDIA GeForce RTX 3070 Ti
RAM	DDR4 64.0GB
Program Language	Python 3.10.11
Library	Cryptography

**Table 6:** 5G-AKA message size.

Message	SUCI	SNN(SN-Name)	5G HE AV	5G SE AV	AUTN	SUPI
Bits	536	256	640	384	128	60

**Table 7:** Communication Overhead.

Protocol	UE (Bits)	Core (Bits)	Total (Bits)
5G-AKA	920	3112	4032
SUCI-AKA	1048	3368	4416
5G-IPAKA	1112	2268	4480
5G-LCCO-AKA	984	3176	4224
5G-AKA${}^{\prime }$	920	3112	4032
EAP-TLS 1.3	5520	5264	10,784
EAP-AKA′-FS	2068	3500	5568
5G-AKA-FS	1048	3368	4416

## Data Availability

No new data were created or analyzed in this study. Data sharing is not applicable to this article.
